# Unusually long-term survival in a feline with Fibrodysplasia ossificans progressiva (FOP-like) disease without *ACVR1* gene mutations

**DOI:** 10.29374/2527-2179.bjvm005726

**Published:** 2026-10-02

**Authors:** Claudia de Aurrecoechea, Sebastián Fernández Barrios, Rody Artigas

**Affiliations:** 1 Facultad de Veterinaria, Universidad de la República, Montevideo, Uruguay.; 2 Policlínico Veterinario, Centro Universitario Regional (CENUR) Litoral Norte, Facultad de Veterinaria, Universidad de la República, Salto, Uruguay.; 3 Unidad Académica de Mejora Animal, Departamento de Producción Animal y Salud de los Sistemas Productivos, Facultad de Veterinaria, Universidad de la República, Montevideo, Uruguay.

**Keywords:** polymyositis, polyarthritis, muscle hypertrophy, heterotopic ossification, gene mutation, polimiosite, poliartrite, hipertrofia muscular, ossificação heterotópica, mutação gênica

## Abstract

Fibrodysplasia ossificans progressiva (FOP) is a rare genetic disorder characterized by heterotopic ossification within the connective tissues that affects the muscles, tendons, and joint capsules. It is an autosomal inherited condition caused by mutations in ACVR1, which is associated with ectopic bone formation. A 5-year-old neutered male outdoor cat, clinically diagnosed with FOP-like disease three years prior, presented for follow-up evaluation owing to an unusually long survival. New blood samples and radiographs were obtained to assess disease progression and investigate the patient's genetic background, with the aim of describing this case's clinical evolution and new genetic findings, and sharing the patient's exceptionally rare long-term survival with peers. Radiographic evaluation revealed severe progression of osteoarticular disease, partial to total resorption of previously deposited mineralized tissue, marked hypertrophy of the limb musculature, and an overall increase in radiographic muscular density. Genetic analysis confirmed the absence of the ACVR1 mutation. This is the first reported case of FOP-like disease in a cat in Uruguay, with no ACVR1 mutation detected and an unusually long survival of more than three years after diagnosis.

## Resumo

A fibrodisplasia ossificante progressiva (FOP) é uma doença genética rara caracterizada por ossificação heterotópica em tecidos conjuntivos, afetando músculos, tendões e cápsulas articulares. É uma condição hereditária autossômica causada por mutações no gene ACVR1, associada à formação óssea ectópica. Um gato macho castrado, com 5 anos de idade e acesso ao ambiente externo, previamente diagnosticado clinicamente com doença semelhante à FOP (FOP-like) há três anos, foi encaminhado para avaliação de acompanhamento devido a uma sobrevida excepcionalmente prolongada. Novas amostras de sangue e radiografias foram obtidas para avaliar a progressão da doença e investigar o perfil genético do paciente, com o objetivo de descrever a evolução clínica e os novos achados genéticos deste caso, e compartilhar com a comunidade científica a rara sobrevida prolongada do paciente. A avaliação radiográfica atual revelou progressão severa da doença osteoarticular, reabsorção parcial a total de tecido previamente depositado mineralizado, hipertrofia acentuada da musculatura dos membros e aumento global da densidade muscular radiográfica. A análise genética confirmou a ausência de mutações no gene ACVR1. Este gato representa o primeiro caso relatado de doença FOP-like em felinos no Uruguai, sem detecção de mutação no gene ACVR1 e com sobrevida incomumente longa de mais de três anos após o diagnóstico.

## Introduction

Fibrodysplasia ossificans progressiva (FOP) is a rare genetic disorder characterized by heterotopic ossification of connective tissues, affecting the muscles, tendons, and joint capsules. It is classified as an ultra-rare disease (Maekawa et al., 2022), but has devastating effects on affected individuals, leading to progressive irreversible tissue hardening. These characteristics were formerly known as the “Stone Man Syndrome” (Hwang et al., 2022). The first feline case was reported in 1980 and was described as an ‘FOP-like condition’ (Elvira, 2010). To date, fewer than 15 feline cases involving animals ranging in age from five months to ten years have been reported worldwide. The majority of these died or were euthanized after diagnosis, except for one case in Brazil that survived for years after diagnosis (Crivelenti et al., 2012). Heterotopic ossification has also been reported in dogs (De Paolo et al., 2022).

Classic FOP is caused by a recurrent activation mutation (c.617G>A; p.R206H) in the *ACVR1* (ALK2) gene. *ACVR1* encodes activin A type I receptor/activin-like kinase 2, which functions as a type I receptor for Bone Morphogenetic Proteins (BMPs). BMPs are growth factors belonging to the transforming growth factor-beta (TGF-β) superfamily. Under normal physiological conditions, BMPs promote the proliferation of the connective tissue cells and activate differentiation factors that induce the transformation of the connective tissue into bone. However, BMP signaling is dysregulated in the presence of this mutation, leading to heterotopic bone formation in the soft tissues (Billings et al., 2007).

Although it has been suggested that inflammation, regardless of its origin, may lead to heterotopic ossification, the precise relationship between *ACVR1* mutations and this process remains unclear (6). Therefore, the mechanisms by which inflammation or flare-ups activate the BMP signaling pathway in patients with FOP are still not fully understood. In humans, flare-ups are associated with intramuscular injections, dental procedures, and viral infections. Interestingly, both humans and felines exhibit heterotopic bone formation in non-essential muscles, such as the myocardium or diaphragm. Notably, an *ACVR1* mutation found in humans with FOP was also identified in cats with FOP. One published report has described two affected feline patients in whom the mutation was genetically confirmed (Casal et al., 2019; Gaschen et al., 2004). Additional cases were diagnosed based on radiographic findings, advanced imaging techniques, or post-mortem histopathological examinations (Klang et al., 2013).

Although no definitive treatment for FOP currently exists, glucocorticoids and nonsteroidal anti-inflammatory drugs (NSAIDs) have been used with varying degrees of success. Promising therapeutic options are currently under investigation in humans, including ongoing phase II and III clinical trials (Meng et al., 2022; Smilde et al., 2022). In Uruguay, FOP has not been previously documented in cats or other animal species, except for a few human case reports (Pérez et al., 2015).

The objective of this report is to describe the clinical evolution, new genetic findings, and share the patient’s exceptionally rare long-term survival with peers.

## Case report

A two-year-old, recently neutered, outdoor male cat was clinically evaluated underwent initial clinical evaluation for walking difficulty (Figure 1). The owners reported that the cat had difficulty jumping, mainly using its left hind limb, and occasionally crossed its hind limbs. As described in a previous report, at that time, the cat was alert and responsive, with normally colored mucous membranas, but showed poor body condition with limb and dorsal neck muscle hypertrophy. Orthopedic evaluation revealed grade 3 mixed lameness (classified as an inability to stand or walk unassisted), stiffness, and hyperextension of the left pelvic limb. Worsening of symptoms was observed in the right pelvic limb. Both pelvic limbs showed alopecic areas that were not associated with overgrooming according to the owner’s history. A FOP-like diagnosis was established based on radiographic and histopathological findings, including hematoxylin-eosin and Masson’s trichrome staining (de Aurrecoechea et al., 2023). Both studies confirmed the presence of heterotopic calcification involving muscles (Figures 2 and 3).

**Figure 1:**
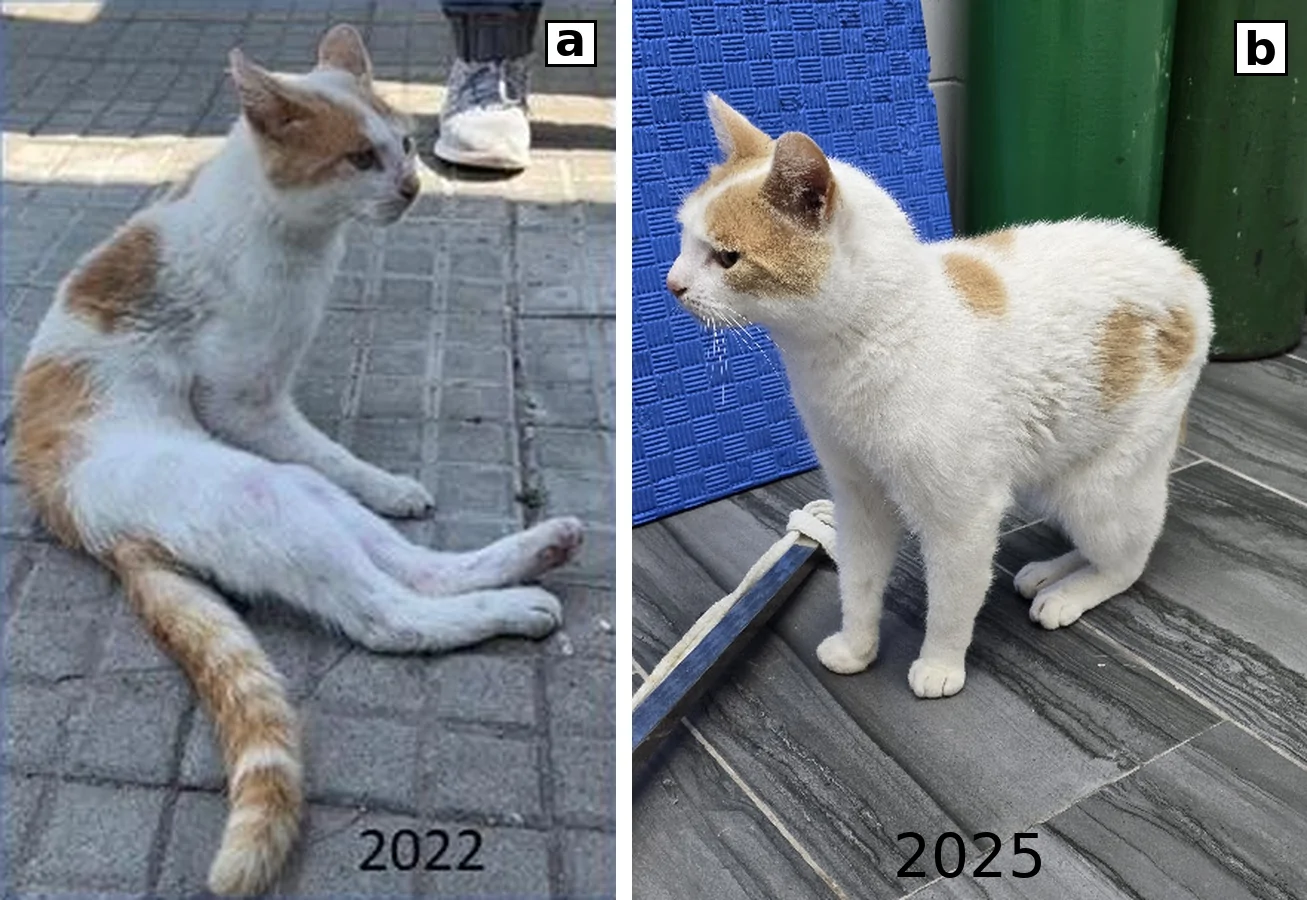
Photographs of the feline patient showing changes in overall clinical condition between 2022 and 2025. (A) Patient in 2022, showing poor body condition, impaired skin and coat quality, and an abnormal sitting posture. (B) Patient in 2025, showing improvement in body weight, skin condition, and coat quality and shine.

**Figure 2:**
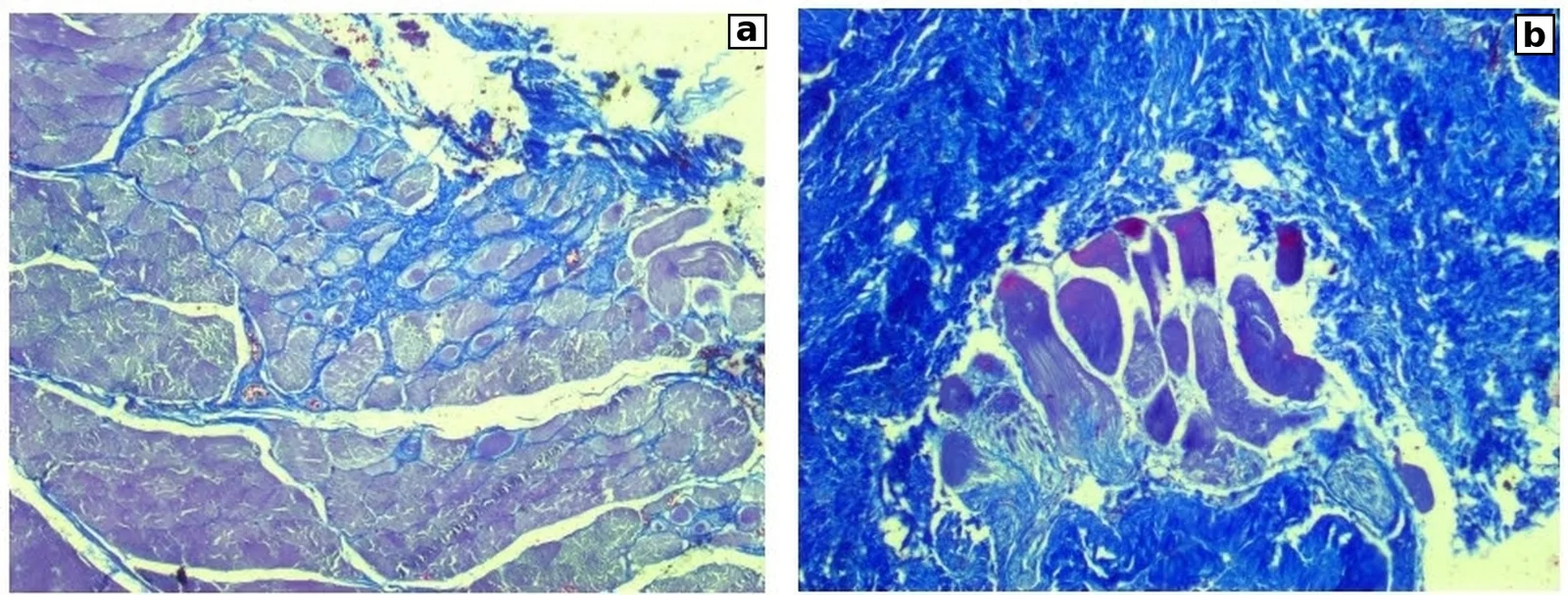
Skeletal muscle biopsy (2022), stained with Masson’s trichrome. (A) Skeletal muscle fascicles separated by increased interstitial and perimysial collagen deposition (blue), consistent with fibrosis. (B) Marked fibrous tissue proliferation (blue), with separation and partial replacement of residual skeletal muscle fascicles. Multifocal areas of mineralized matrix, histologically compatible with heterotopic ossification, were identified within the fibrous connective tissue. Original magnification, 10×.

**Figure 3:**
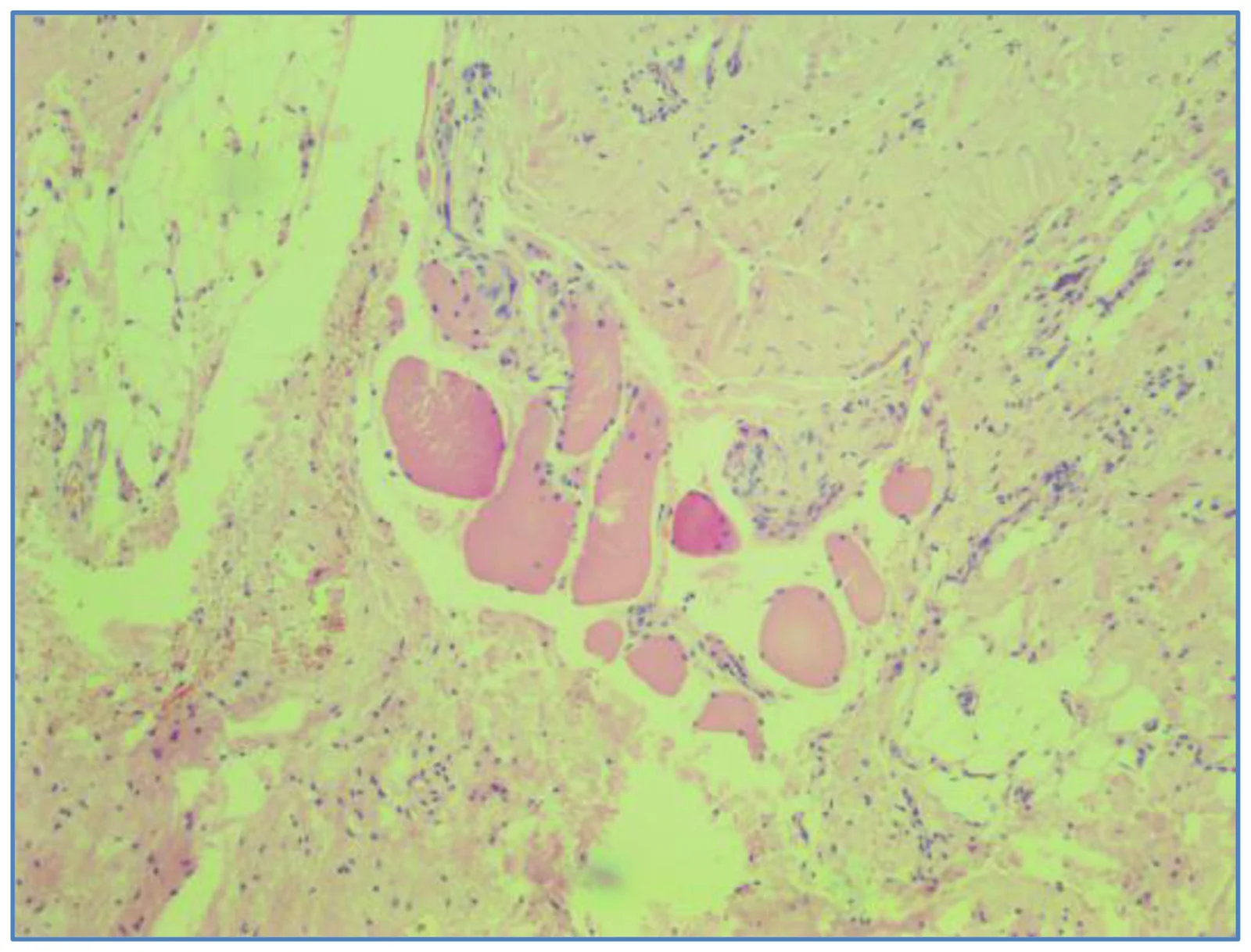
Skeletal muscle biopsy (2022), 10 x, stained by hematoxylin-eosine, showing the calcified muscle tissue as pink.

A genetic study was also conducted during the diagnostic workup, but was not reported at that time. Whole blood samples were collected in EDTA to test for a previously reported *ACVR1* mutations. The samples were sent to the Academic Unit of Animal Genetics, Faculty of Veterinary Medicine, Universidad de la República, Uruguay, Brazil. DNA was extracted from 200 µL of blood using a Genomic DNA Purification Kit (Fermentas, Thermo Fisher, Canada), following the manufacturer’s instructions. PCR amplification of the *ACVR1* fragment containing the mutation site was performed using the MultiGene II thermal cycler (Labnet Inc. Global) in a final reaction volume of 25 µL. The reaction mix included 100 ng of genomic DNA, 2 µL of each primer (10 pmol/µL), 12.5 µL of ImmoMix (Bioline, Australia), and Milli-Q water to complete the volume of the reaction. The PCR protocol included an initial denaturation step at 95°C for 10 min, followed by 35 cycles of denaturation at 94°C for 40 s, annealing at 65°C for 30 s, and extension at 72°C for 1 min, with a final extension step at 72°C for 10 min. Amplicons were analyzed by electrophoresis on a 1.5% agarose gel, stained with GoodView (SBS Genetech Co., Ltd.). The primers used were those described by Casal et al. (2019). F: 5´-ACTACTATCCCTCAGCAAAACCT-3´R: 5´-CAAACGCCTACTCACCTCAGAT-3.’ PCR amplification and sequencing of the fragment did not reveal the G>A substitution at codon 206 (Figure 4).

**Figure 4:**
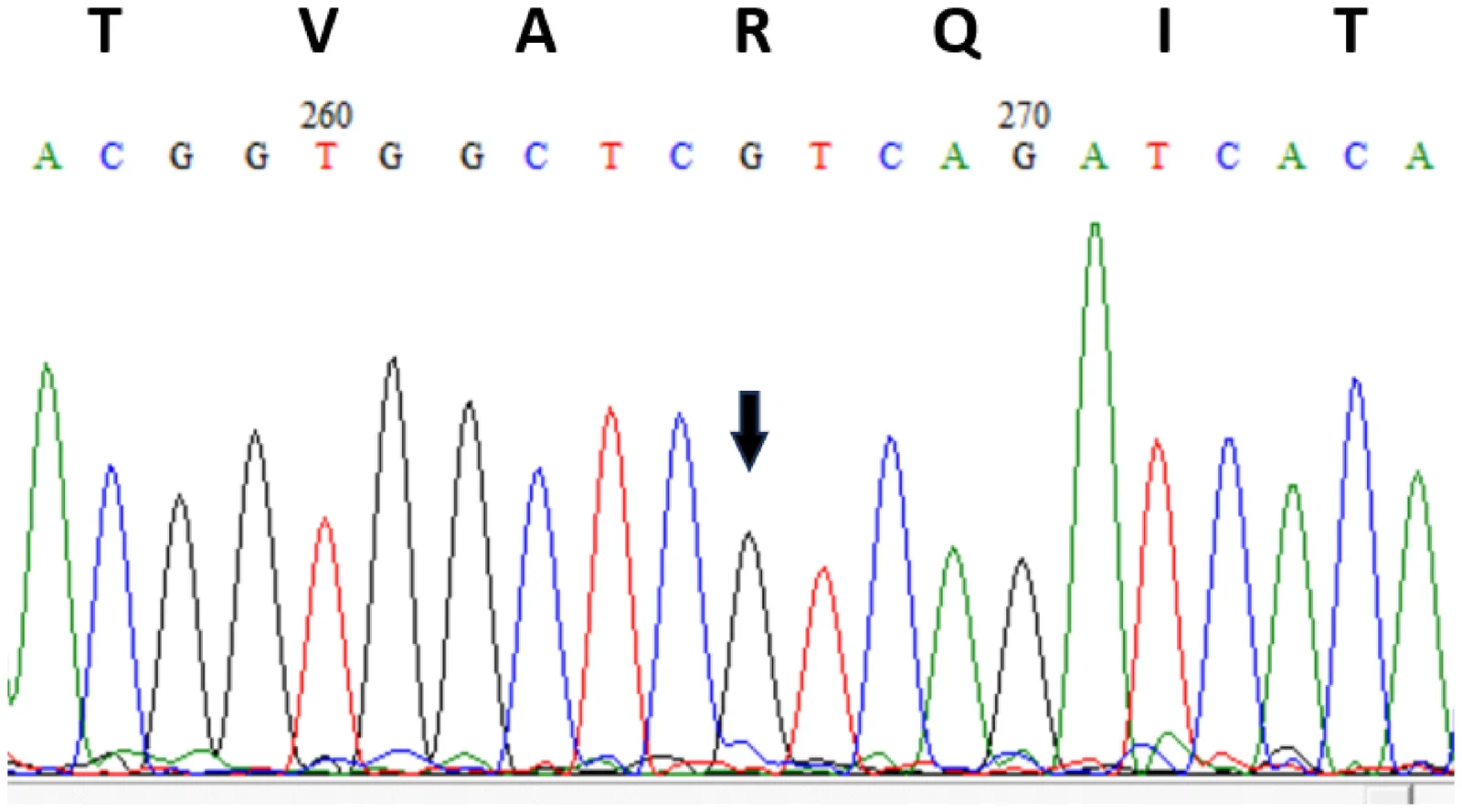
Electropherogram of the amplified fragment of the *ACVR1* gene. The black arrow indicates the site of the mutation, showing that the animal is a normal homozygous.

Three years (2025) after the patient’s initial clinical evaluation, owing to the unusually long-term survival of this patient, new blood samples and radiographs were obtained for follow-up examination. Complete blood count, serum biochemistry, serum calcium levels, and thyroid hormone levels were all within the normal limits. Immunochromatographic tests for feline leukemia virus (FeLV) antigens and antibodies against feline immunodeficiency virus (FIV) were negative, as was the PCR test for FeLV. On the current radiographs, an increase in the size and radiopacity of the musculature of the thoracic and pelvic limbs was observed, along with partial to total resorption of the previously identified mineralized material. Severe signs of osteoarthritis were noted in the stifle joints, including ankylosis, increased density of the subchondral bone tissue, and the presence of large osteophytes, which were more severe in the right stifle joint. Separation and abduction of both pelvic limbs were not possible owing to hip and stifle ankyloses. Severe osteoarthritic changes were observed in the right coxofemoral joint (Figures 5, 6, 7, 8, 9). However, the feline’s overall clinical condition improved compared with the previous evaluation, particularly the skin condition and body weight. The cat had regained the ability to groom itself, which was not possible at the previous clinical assessment. Despite the worsened radiological findings, specifically the osteoarticular changes, the cat’s quality of life remained acceptable, with no evidence of chronic pain. The degree of lameness remained almost unchanged, with a persistent inability to flex the stifle joints and neck; however, mild improvement in climbing was observed. The cat adopted an unusual sitting posture and supported its weight on the metatarsus.

**Figure 5:**
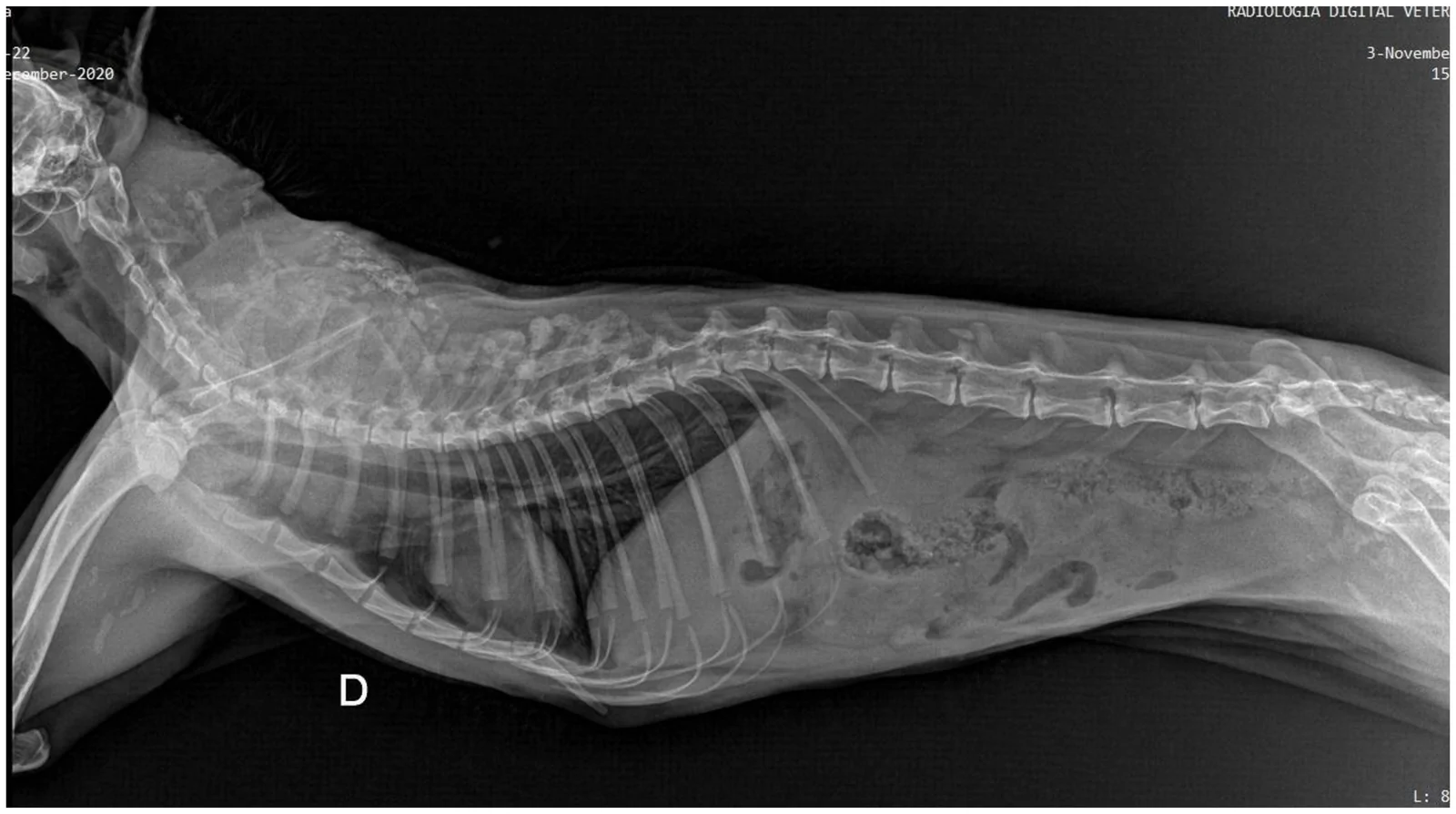
Lateral radiographic view showing evident mineralization in the region of the **brachiocephalicus**, **longissimus dorsi**, and **biceps brachii** muscles (2022).

**Figure 6:**
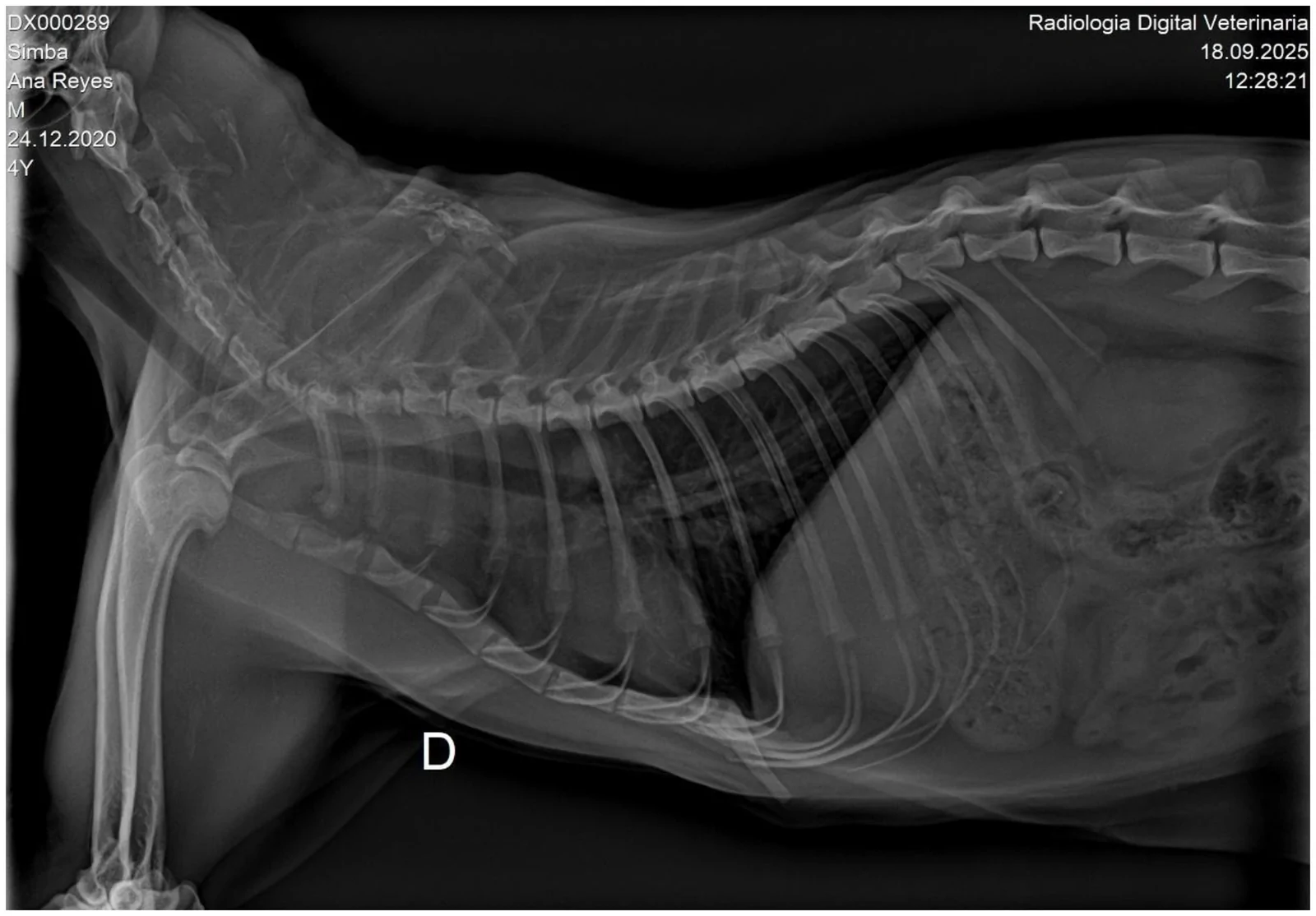
Lateral radiographic image of the same affected area showin in Figure 2, demonstrating lesser calcification but increased muscular density (2025). D = right side.

**Figure 7:**
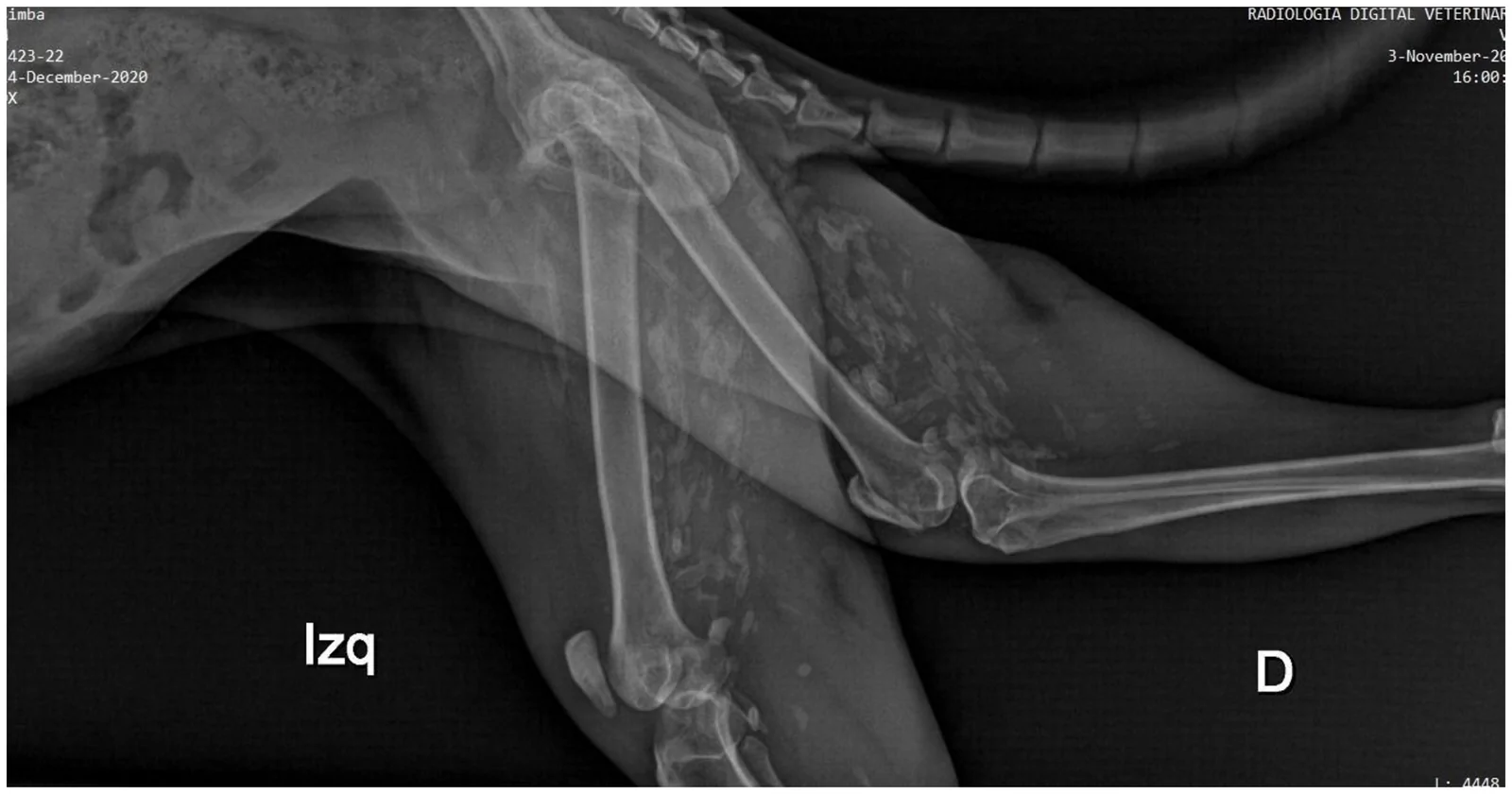
Laterolateral radiographic projection of the pelvic limbs demonstrating marked mineralization of the ischiotibial musculature (2022). D = right side; Izq = left side.

**Figure 8:**
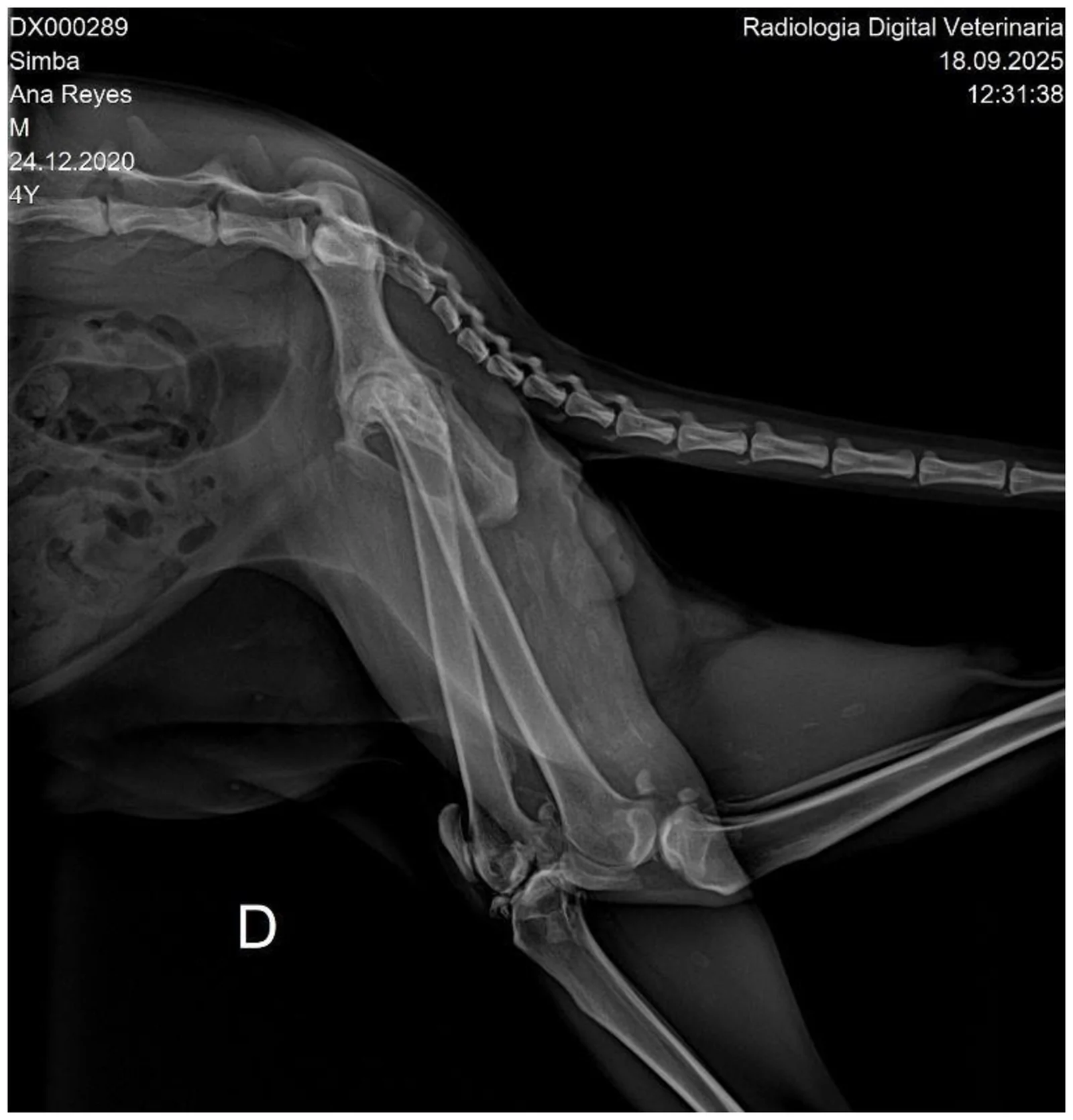
Lateral radiographic image of the pelvic limbs showing the inability to abduct or separate the limbs, as well as osteoarthritis of the stifle and coxofemoral joints, as well as as persistent spread-out calcification of the ischiotibial musculature (2025). D = right side.

**Figure 9:**
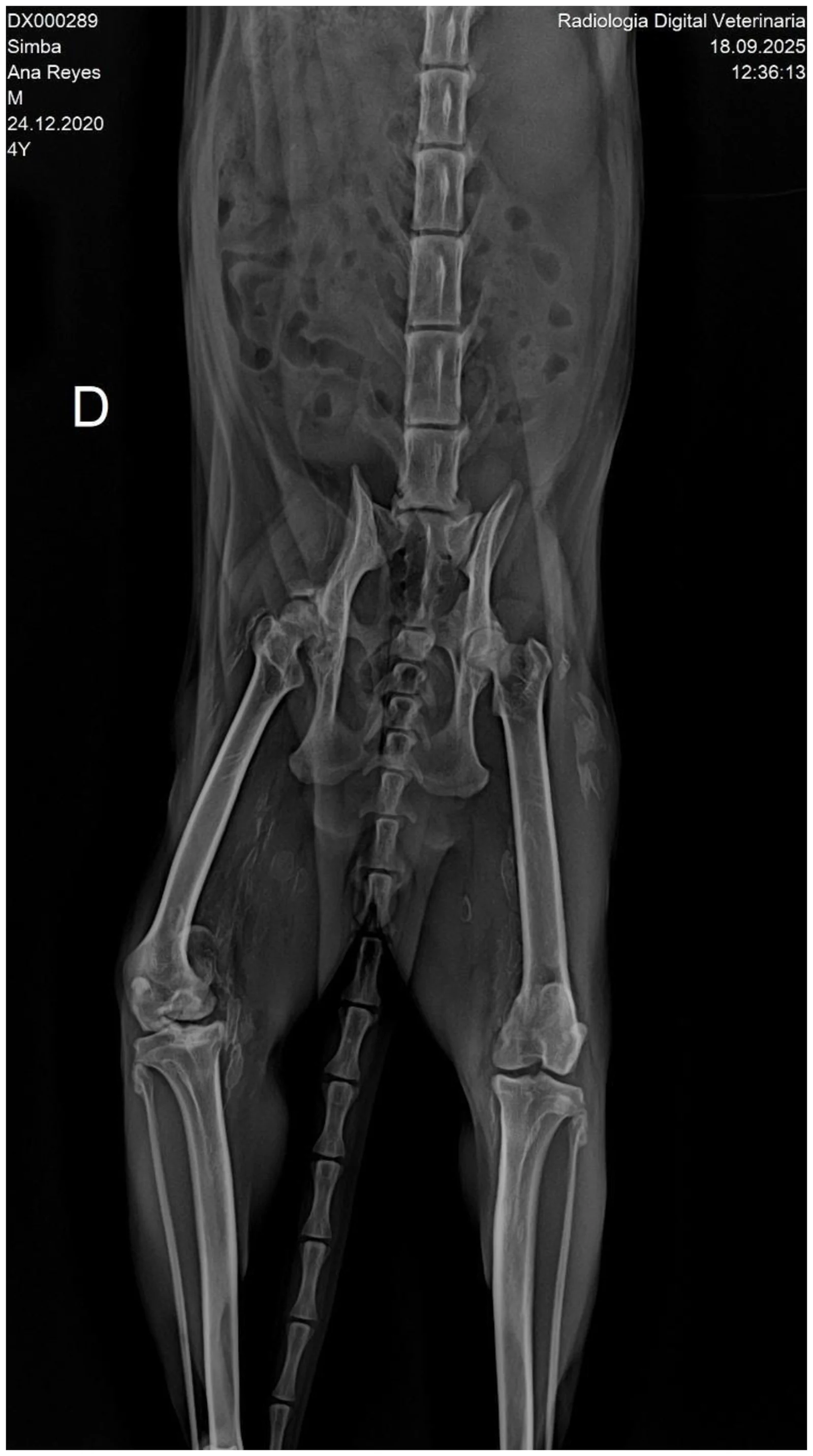
Dorsoventral radiographic image showing the remaining calcification of the pelvic limbs, with severe arthrosis of the right stifle and right coxofemoral joints (2025). D = right side.

Although the owner reported no evidence of pain and the authors observed an overall improvement in the cat’s general condition, the radiographic progression and severity of osteoarticular changes supported the consideration of the underlying chronic pain in this case. Therefore, chronic pain management has been prescribed by established consensus recommendations, emphasizing careful selection and monitoring of long-term NSAID therapy to optimize the quality of life, while minimizing adverse effects in feline patients (Smilde et al., 2022; Steagall et al., 2022; Taylor et al., 2024). In this particular case, treatment consisted of meloxicam, tramadol, and gabapentin, and physical therapy was suggested to maintain an acceptable quality of life.

## Discussion

To date, there have been no documented cases of FOP-like disease in felines or other non-human species in Uruguay, making this case a unique addition to the limited number of reports worldwide. The patient, who underwent initial evaluation at 2 years of age, remained alive at the time of reporting, similar to a patient reported in Brazil (Cita Brazil), who manifested similar patterns of pelvic limb mineralization. However, these authors performed surgery to eliminate the calcified tissue, with poor results and mineralization recurrence. According to previously published cases, no clear correlation with age has been identified, as affected individuals range in age from only a few months to 10 years (Asano et al., 2006; Valentine et al., 1992; Warren & Carpenter, 1984). Therefore, the age of the patient in the present case was within the range reported in the literature. Given the limited number of feline cases, determining a sex predisposition for this condition remains difficult.

Notably, in two previously reported cases of digital malformations, the same mutation responsible for BMP pathway activation in humans was identified in cats (Casal et al., 2019). Specifically, a heterozygous mutation in *ACVR1* appeared to be a determining factor in these cases (Chan et al., 2023). In the present case, the mutation previously reported by Casal et al. (2019) was not observed. However, the possibility of other mutations within different regions of *ACVR1* or in metabolically related genes cannot be excluded. This is relevant because allelic heterogeneity is a well-known feature of hereditary disorders (Woodward et al., 2022). Therefore, additional genetic analyses, including sequencing of the entire *ACVR1* coding region and the assessment of other functionally related genes, are warranted to determine whether alternative variants are present in this patient.

The radiological findings in this case, with lesions localized in the hindlimbs and additional involvement of the cervical region, thoracic spine, and shoulder areas, are consistent with those described in prior reports. Previous studies have noted soft tissue lesions with calcified areas in the muscles of the cervical and thoracic spine, shoulders, and hind limbs (Smilde et al., 2022; Yabuzoe et al., 2009). However, given the unusually long survival time in the present patient, it was not possible to compare the current radiographic findings, particularly the apparent resorption of mineralized tissue, with any previously reported feline cases, as most of the affected cats died or were euthanized earlier in the course of the disease. Therefore, similar radiographic changes may have occurred in other cases, but could not be documented owing the shorter follow-up period.

Given the overlapping clinical and radiographic presentations of periarticular or intramuscular mineralization, several conditions should be considered in the differential diagnosis of FOP-like disease in companion animals. Among these, myositis ossificans circumscripta is a nonhereditary, typically localized form of heterotopic ossification most commonly triggered by trauma or intramuscular injection. Unlike FOP-like disease, it tends to remain solitary and non-progressive, rather than involving multiple muscle groups in a bilaterally symmetric, progressive pattern (Klang et al., 2013). Calcinosis circumscripta, an idiopathic, dystrophic, or metastatic mineralization syndrome reported predominantly in young, large-breed dogs, can usually be distinguished histologically by the identification of well-circumscribed lakes of granular basophilic minerals surrounded by foreign-body giant cells and histiocytic inflammatory reactions; this pattern contrasts with the endochondral ossification, cartilaginous metaplasia, and marrow formation within thickened fibrous connective tissue characteristics of FOP-like lesions (Klang et al., 2013; Tafti et al., 2005). Metastatic and dystrophic calcification secondary to hypervitaminosis D, chronic kidney disease, or hyperadrenocorticism-associated calcinosis cutis is generally accompanied by identifiable underlying metabolic or renal derangement and abnormal serum calcium, phosphorus, or vitamin D concentrations, features that were excluded in the present case owing to the normal serum biochemistry and calcium levels (De Luca & Del Piero, 2026). Finally, a FOP-like phenotype has also been described in dogs, independent of the *ACVR1* mutation identified in cats (De Paolo et al., 2022; Guilliard, 2001), reinforcing the idea that molecular confirmation, when available, remains the most specific criterion for distinguishing true FOP-like disease from other heterotopic ossification syndromes, even though its absence, as in the present case, does not rule out the diagnosis.

Unlike previously reported cases, our patient did not exhibit any lesions or events commonly associated with vaccination-induced flare-ups. In such instances, muscular pain typically develops within 48 hours of annual vaccination (Elvira, 2010). Although immune-mediated flare-ups have been documented in humans, no clear evidence of an immunological trigger was identified in the present case. Nevertheless, given the outdoor lifestyle of cats, the definitive exclusion of potential environmental or infectious triggers remains difficult.

Biopsies from both sampled sites revealed the same lesion pattern, with no inflammatory infiltrates, suggesting different disease progression stages. The absence of a preceding inflammatory process has also been noted in human cases without overt flare-up episodes.

Although this cannot be directly verified, it may be hypothesized that the owners’ personal history and empathic disposition toward individuals with disabilities contributed to the exceptional long-term care provided to the present patient. Studies on companion animal care have reported that individuals with special needs often exhibit heightened empathy, anthropomorphism, and a strong caregiving identity, which may facilitate sustained care efforts and a nonjudgmental attitude towards disability (Katz & Burchfield, 2018).

## Conclusions

To our knowledge, this is the first documented case of FOP-like disease in a cat in Uruguay. The patient remained alive, exhibiting only signs of muscular disease, grade 3 lameness, stifle joint hyperextension due to soft tissue calcification, and a permanent inability to ventroflex the neck. However, despite these limitations, the animals’ quality of life was considered acceptable. A specific *ACVR1* mutation identified in previously reported feline cases was not detected in this patient, suggesting allelic heterogeneity in FOP-like conditions in cats.
